# Cyanobacterial blooms contribute to the diversity of antibiotic-resistance genes in aquatic ecosystems

**DOI:** 10.1038/s42003-020-01468-1

**Published:** 2020-12-04

**Authors:** Qi Zhang, Zhenyan Zhang, Tao Lu, W. J. G. M. Peijnenburg, Michael Gillings, Xiaoru Yang, Jianmeng Chen, Josep Penuelas, Yong-Guan Zhu, Ning-Yi Zhou, Jianqiang Su, Haifeng Qian

**Affiliations:** 1 College of Environment, Zhejiang University of Technology, 310032 Hangzhou, People’s Republic of China; 2 Institute of Environmental Sciences (CML), Leiden University, 2300 RA, Leiden, The Netherlands; 3 National Institute of Public Health and the Environment (RIVM), Center for Safety of Substances and Products, P.O. Box 1, Bilthoven, The Netherlands; 4 Department of Biological Sciences, Macquarie University, Sydney, NSW 2109 Australia; 5 Key Laboratory of Urban Environment and Health, Institute of Urban Environment, Chinese Academy of Sciences, 361021 Xiamen, People’s Republic of China; 6 CSIC, Global Ecology Unit CREAF-CSIC-UAB, Bellaterra, 08193 Barcelona, Catalonia Spain; 7 CREAF, Cerdanyola del Vallès, 08193 Barcelona, Catalonia Spain; 8 State Key Lab of Urban and Regional Ecology, Research Center for Ecoenvironmental Sciences, Chinese Academy of Sciences, 100085 Beijing, People’s Republic of China; 9 State Key Laboratory of Microbial Metabolism, and School of Life Sciences & Biotechnology, Shanghai Jiao Tong University, 200240 Shanghai, People’s Republic of China

**Keywords:** Water microbiology, Microbial ecology

## Abstract

Cyanobacterial blooms are a global ecological problem that directly threatens human health and crop safety. Cyanobacteria have toxic effects on aquatic microorganisms, which could drive the selection for resistance genes. The effect of cyanobacterial blooms on the dispersal and abundance of antibiotic-resistance genes (ARGs) of concern to human health remains poorly known. We herein investigated the effect of cyanobacterial blooms on ARG composition in Lake Taihu, China. The numbers and relative abundances of total ARGs increased obviously during a *Planktothrix* bloom. More pathogenic microorganisms were present during this bloom than during a *Planktothrix* bloom or during the non-bloom period. Microcosmic experiments using additional aquatic ecosystems (an urban river and Lake West) found that a coculture of *Microcystis aeruginosa* and *Planktothrix agardhii* increased the richness of the bacterial community, because its phycosphere provided a richer microniche for bacterial colonization and growth. Antibiotic-resistance bacteria were naturally in a rich position, successfully increasing the momentum for the emergence and spread of ARGs. These results demonstrate that cyanobacterial blooms are a crucial driver of ARG diffusion and enrichment in freshwater, thus providing a reference for the ecology and evolution of ARGs and ARBs and for better assessing and managing water quality.

## Abstract (web summary)

Qi Zhang, Zhenyan Zhang et al. show that the numbers and relative abundance of antibiotics-resistance genes increase during a cyanobacterial bloom in Lake Taihu of China. This correlation provides insights to the ecologists and the policy makers who manage the quality of fresh water.

## Introduction

Eutrophication, which is driven by increased inputs of nutrients from intensified agriculture, frequently induces cyanobacterial blooms, especially when coupled with warming^1^. Such blooms are increasingly frequent in freshwater and marine environments; 30–40% of the world’s lakes and reservoirs and nearly 80% of the freshwater bodies in China are eutrophic^2^. Cyanobacterial blooms decrease water quality and adversely affect the functioning of aquatic ecosystems^3–7^, which can alter the bacterial community structure and disrupt recreation and human health^8,9^. Unique microbial communities are assembled during a cyanobacterial bloom by stochastic and potentially deterministic processes, including competition, mutualism, and trade-offs^10^.

Antibiotic resistance is one of the most pressing global public health concerns^11–13^. Antibiotic-resistance genes (ARGs) are emerging environmental pollutants^14^, which can be transferred between antibiotic-resistant bacteria (ARBs) and non-antibiotic resistant bacteria via numerous mobile genetic elements (MGEs)^15,16^, are becoming more prevalent in various water compartments, including surface water, drinking water, sewage, and natural waters^17–19^. Human activities such as the discharge of domestic sewage and the fertilization of soil by the addition of human and animal feces with subsequent leaching^17,18,20^ cause freshwater ecosystems to become an important repository for ARGs^21^. Some evidence suggests that ARGs carried by the ARBs are being transferred from the environment to organisms in the human body, and the transfer of clinical ARGs to environmental microorganisms is a topic of concern. Various studies have reported that humans can be infected by ARBs by direct contact with natural waters, including ingestion of aquatic products, drinking water, and during swimming^22–24^. The emergence and distribution of ARGs in aquatic ecosystems such as rivers, lakes and sewage are subject of ongoing research^17,25^ and studies indicated that changes in bacterial communities are a key driver underlying ARG composition in nature waters^12,18,22,25^. Since the structure and composition of bacterial communities are affected by cyanobacterial blooms^26,27^, ARG composition is likely to strongly be shaped in response to cyanobacterial blooms. However, to the best of our knowledge, the role of biotic and abiotic processes such as competition and environmental filtering in assembling ARB communities as well as resistome (ARG groups) during cyanobacterial blooms is remained unknown.

To shed light on the impacts of cyanobacterial blooms on the emergence and spread of ARGs, the different periods of cyanobacterial bloom were selected as the peak stages (July and August 2016), decline stage (September 2016) and non-bloom stage (March and May 2017) in the present study according our previous study^28^. We monitored the microbial community and the composition of ARGs in Lake Taihu, a natural freshwater lake that is eutrophic and has annual cyanobacterial blooms, during these periods by sequencing amplicons of the 16S ribosomal RNA (rRNA) gene and the internal transcribed spacer (ITS) as well as the high-throughput quantitative PCR (HT-qPCR). In addition, laboratory co-culturing experiments using additional aquatic ecosystems (urban river and Lake West) then explored whether the cyanobacteria were a crucial driver to the emergence and dissemination of ARGs. Laboratory microcosmic investigations have been widely used to identify the changes in diversity and composition of ARGs and the response of MGEs to inputs of xenobiotic pollutants in the previous studies^29–32^.

By the field works and laboratory experiments, we aimed to (1) monitor the changes of ARG composition throughout different cyanobacterial bloom periods in Lake Taihu; (2) reveal the contribution of biotic and abiotic factors to the emergence and spread of ARGs in freshwater; and (3) explore whether different species of cyanobacteria mediate various freshwater resistomes. Strong interactions between the ARGs, microbial assemblages and the cyanobacterial blooms need to be understood in the broader context of managing ARGs in aquatic ecosystems.

## Results

### Environmental conditions and ARGs at different stages of cyanobacterial blooms

Physicochemical parameters of water (temperature, pH and TN, TP, NO_3_^−^, and NH_4_^+^ concentrations) were documented during the three phases of the cyanobacterial bloom in Lake Taihu (Fig. S1a). The data from 16S rRNA gene (hereafter 16S) sequencing and qPCR indicated that the dominant cyanobacterial species were *Planktothrix* in July and *Microcystis* in August, respectively (Fig. S1b).

**Fig. 1:**
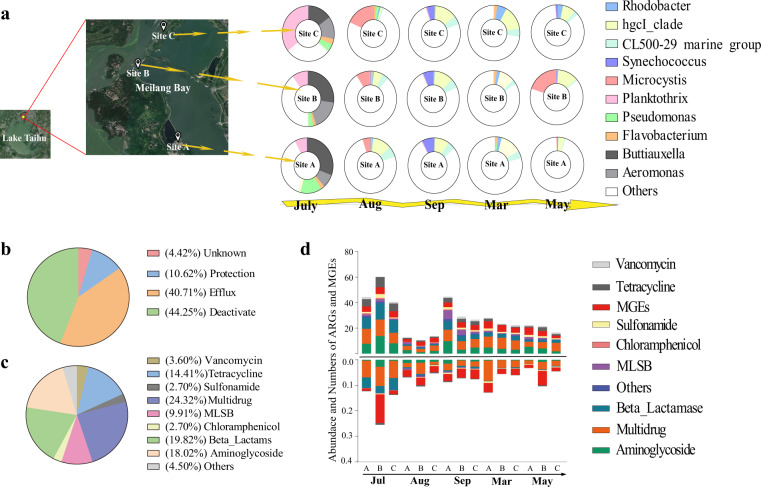
Bacterial and antibiotic resistance genes (ARGs) composition in different stages of cyanobacteria blooms in Lake Taihu. Satellite map of the three sampling sites (generated by Google Maps) and the relative abundances of the ten most common genera in the bacterial community in each sampling month and site (**a**). ARG classifications based on antibiotic resistance mechanisms (**b**) and the antibiotic classes to which they confer resistance (**c**). Abundances and numbers of ARGs associated with various antibiotics and MGEs during the cyanobacterial blooms (*n* = 4) in Lake Taihu (**d**).

Totals of 122 ARGs and nine MGEs were detected in the samples. They were classified into eight categories, aminoglycoside, beta-lactams, macrolide-lincosamide-streptog-ramin B (MLSB), multidrug, fluoroquinolone, tetracycline, vancomycin, and others based on the classes of resistance to antibiotics. The ARGs encompassed four mechanisms of antibiotic resistance, antibiotic deactivation (44.2%), efflux pumps (40.7%), cellular protection (10.6%) and unknown (4.5%) (Fig. 1b), and covered several major classes of antibiotics, including those involved in multidrug resistance (24.3%), beta-lactams (19.8%), aminoglycosides (18%) and tetracycline (14%) (Fig. 1c). The numbers of ARGs ranged from 10 to 66, with most observed in July and fewest in August (Fig. 1d, one-way ANOVA, *P* < 0.05). Multidrug-resistance genes were the most abundant ARG category in almost all samples, in agreement with a recent study reporting that multidrug-resistance gene was the most abundant ARGs in Lake Taihu sediments^33^.

### Relationships among the Lake Taihu ARGs, MGEs, and microbial communities

We sequenced the 16S and ITS amplicons to analyze the formation of the cyanobacterial bloom and the composition of the microbial community in Lake Taihu. Totals of 1,957,979 (bacterial) and 1,643,396 (fungal) high-quality sequences were obtained by assembling and quality filtering, with 27,593–60,812 bacterial samples and 28,499–46,939 fungal samples sequenced. A Procrustes analysis indicated that the abundance of lake bacterial operational taxonomic units (OTUs) was strongly correlated with ARG abundance, with a significant goodness-of-fit (*M*^2^ = 0.503, *P* < 0.01, 9999 permutations) based on the Bray–Curtis dissimilarity metric (Fig. S1c). The Mantel test also indicated a significant positive correlation between the abundance of bacterial OTUs and the ARG data across all monthly samples (*r* = 0.68, *P* = 0.01). Both the Procrustes and Mantel analyses indicated that fungal abundance was not correlated with ARG abundance, as expected (*M*^2^ = 0.73, *r* = 0.03, *P* = 0.32, 9999 permutations) (Fig. S1d).

We thus focused on the relationship between bacterial genera and the abundances of ARGs and MGEs. The abundances of the ten most common bacterial taxa across all sample sites and times are shown in Fig. 1a. Abundant taxa generally belonged to the Proteobacteria or Cyanobacteria. Samples collected in July contained abundant Proteobacteria in the genera *Pseudomonas*, *Buttiauxella*, and *Aeromonas*.

**Fig. 2:**
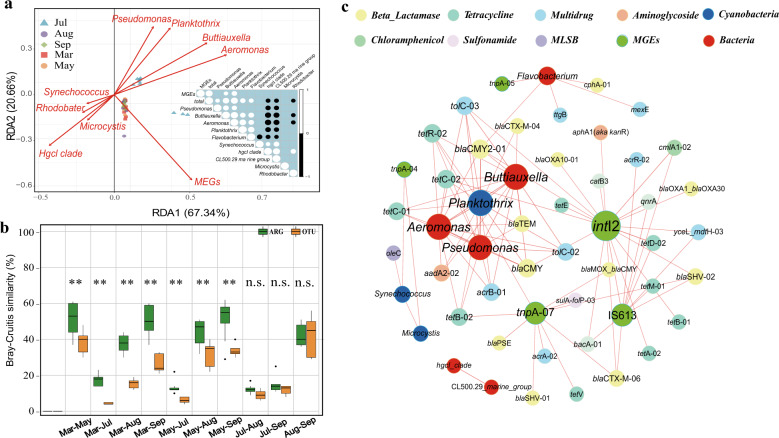
Relationships between ARGs and bacterial communities in Lake Taihu. An RDA analysis was used to determine the correlations among the abundances of ARGs, bacteria, cyanobacteria, and MGEs. The ARGs of different samples are represented by dots with different colors, and bacteria, cyanobacteria and MGEs are represented by arrows and the inner panel shows the correlations among bacteria and MGEs (**a**). Bray−Curtis similarity of the bacterial communities and ARG compositions between each sampling month (**b**). **indicates significant differences between the bacterial communities and ARG compositions at *P* < 0.01 by a one-way ANOVA, and n.s. indicates not significant. Network analysis identifying the patterns of co-occurrence among the ARGs, MGEs and bacteria (top 10 genera) with significant correlations (**c**). The size and color of the nodes represent the linking numbers of patterns and the classes of the ARGs, MGEs and bacteria, respectively. The width of the line represents the strength of the correlation. Only strong (Pearson, *r* > 0.6) and significant (*P* < 0.05) correlations are shown.

A redundancy analysis found that the two first axes explained 88% of the total variation (Fig. 2a). The abundances of the genera *Pseudomonas*, *Planktothrix*, *Buttiauxella* and *Aeromonas* were positively correlated with ARG composition in July. In particular, MGE abundance was most strongly correlated with ARG abundance and most weakly correlated with *Planktothrix* abundance. Genes conferring multidrug resistance and genes coding for beta-lactamases were the ARGs that changed the most during the different bloom stages, and their abundances were significantly positively correlated with the abundances of the dominant genera (*Pseudomonas*, *Planktothrix*, *Buttiauxella*, *Aeromonas* and *Flavobacterium*) (Pearson, *r* > 0.6, *P* < 0.05). The abundances of the other two cyanobacterial genera were not significantly correlated with ARG abundance (Pearson, *r* > 0.6, *P* < 0.05), indicating a strong association between *Planktothrix* and the emergence of ARGs.

### Different cyanobacterial blooms are associated with different ARG compositions in Lake Taihu

A principal coordinate analysis (PCoA) based on Bray–Curtis distances found that the antibiotic resistome in July was dramatically separated from ARG composition in the other months (Fig. S1e). The ARG compositions were most similar during May and March and least similar between July and March (Fig. 2b), indicating a larger change in the antibiotic-resistant community between the bloom and non-bloom months than within the non-bloom months. The change in the antibiotic-resistant communities between the samples, however, was weaker than the change in the bacterial communities using Bray–Curtis similarity, suggesting that the resistome was relatively stable.

We used network analysis to identify the patterns of co-occurrence among the ARGs, MGEs (including seven transposases and two integron-integrase genes) and bacteria (10 most common genera) (Fig. 2c). *Planktothrix* abundance was strongly positively correlated with the abundances of the dominant genera *Buttiauxella*, *Aeromonas*, *Pseudomonas* and *Flavobacterium*, and the abundances of ARGs associated with *Planktothrix* were significantly correlated with the abundance of these bacteria (Pearson, *R* > 0.6, *P* < 0.05). This finding indicated that *Planktothrix* may influence the resistome indirectly by promoting the growth of species that contain ARGs. The abundances of major MGEs, such as *intI2*, *tnp*A-07 and *IS*613, were strongly correlated with the abundances of most ARGs (Pearson, *R* > 0.6, *P* < 0.05), implying that these MGEs were physically linked to the ARGs.

### Verification of the relationship between ARGs and cyanobacteria in a coculture

**Fig. 3:**
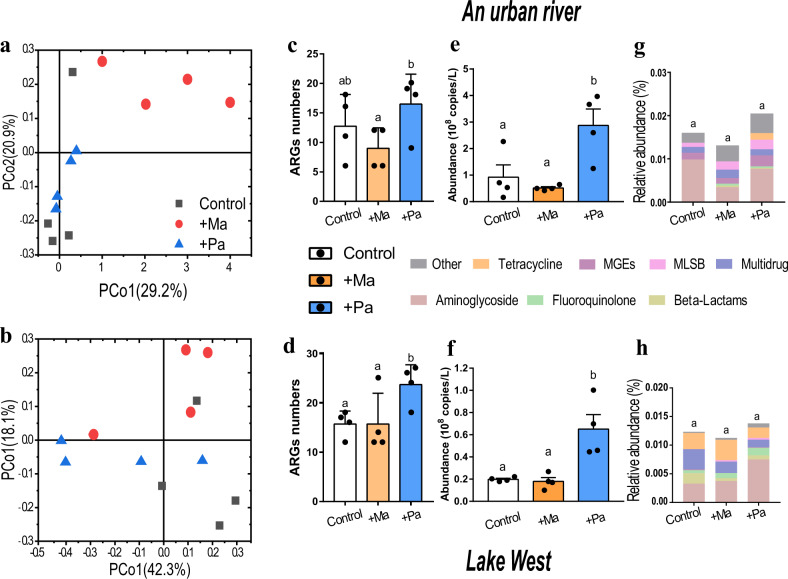
Composition and diversity of ARGs in the different co-culture systems, including the urban river and Lake West cocultured with *Microcystis aeruginosa* (+Ma) and *Planktothrix agardhii* (+Pa). Principal coordinate analysis (PCoA) of ARG communities in an urban river (**a**) and Lake West (**b**) using Bray−Curtis distances. The numbers of detected ARGs in urban river (**c**) and Lake West (**d**). The absolute abundances of ARGs in urban river (**e**) and Lake West (**f**) and relative abundances of various types of ARGs in urban river (**g**) and Lake West (**h**). Different letters indicate significant differences between the control and treatments (*P* < 0.05, one-way ANOVA). Data from four replicates (*n* = 4) are represented as means ± SD.

We tested whether cyanobacteria could affect ARG composition in other aquatic environments by coculturing *M. aeruginosa* and *P. agardhii* in two representative natural water bodies, the urban river and Lake West. A PCoA using Bray−Curtis distances based on the relative abundances of ARGs found a significant separation between cocultures with added *M. aeruginosa* and those with added *P. agardhii* in the urban river samples (Adonis analysis, *R* = 0.406, *P* = 0.035) (Fig. 3a). A similar trend was observed in water originating from Lake West, although the trend was not significant (Adonis analysis, *R* = 0.188, *P* = 0.174) (Fig. 3b). ARG numbers were significantly higher in the *Planktothrix* treatments than the control and the *Microcystis* groups (one-way ANOVA, *P* < 0.05), regardless of whether the cyanobacteria were cocultured in water from the urban river or Lake West, and were significantly lower in the *Microcystis* treatments (one-way ANOVA, *P* > 0.05) (Fig. 3c, d).

The average absolute abundances of the ARGs (number/L) were significantly higher in the *Planktothrix* treatments than the control and *Microcystis* groups (one-way ANOVA, *P* < 0.05) (Fig. 3e, f). Similarly, the relative abundances of the ARGs (ARG/16S number) of the *Planktothrix* treatments was highest among all treatments in the cocultured systems (urban river and Lake West) (Fig. 3g, h).

**Fig. 4:**
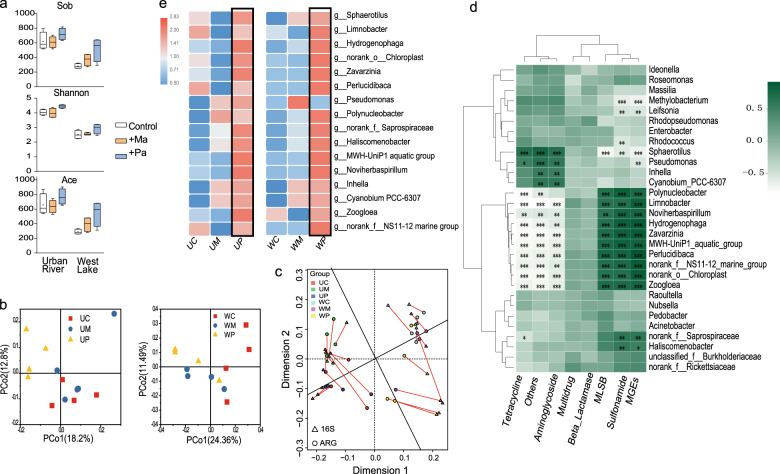
Diversities of bacterial communities and correlations between ARG abundance and the bacterial communities. Alpha diversities (Sob, Shannon, and Ace indices) of bacterial communities in the co-culture systems, including the urban river and Lake West co-cultured with *Microcystis aeruginosa* (+Ma) and *Planktothrix agardhii* (+Pa) (**a**). Principal coordinate analysis (PCoA) of bacterial communities in urban river and Lake West using weighted Unifrac distances (**b**). Correlations between ARG abundance and the bacterial communities were determined by Procrustes and Mantel analyses (**c**). Heatmap showing the connections between the abundances of the ARGs and the bacterial communities at the genus level (30 most common) (**d**). The urban river and Lake West cocultured with *Microcystis aeruginosa*, *Planktothrix agardhii* and control were defined as groups: UM, UP and UC and WM, WP and MC, respectively. The relative abundances of the genera (correlated with the ARGs) among the treatments (*n* = 4) (**e**). *, **, and *** indicate significant differences between the bacterial communities and ARG composition at *P* < 0.05, *P* < 0.01 and *P* < 0.001, respectively, by a one-way ANOVA. Data from four replicates (*n* = 4) are represented as means ± SD.

A total of 1,121,479 sequences were obtained from the 24 samples of laboratory experiment, and 2344 representative 16S OTUs were clustered for downstream analysis. The urban river had more complex and more diverse bacterial communities; the samples from the urban river contained more OTUs and had a higher alpha diversity (Sobs, Shannon, and Ace indices) than samples from Lake West (Fig. 4a). These results also indicated that the *P. agardhii* cocultured system increased the alpha diversity, whereas *M. aeruginosa* had no effect on alpha diversity. Proteobacteria and Bacteroidetes were the main phyla (>10%) in the two natural water bodies, and their abundances did not change significantly in either treatment (Fig. S2) (one-way ANOVA, *P* > 0.05). The PCoA analysis indicated that the bacterial communities of the urban river samples clustered separately after the different treatments (Adonis analysis, *R* = 0.22, *P* = 0.03). Beta diversity did not differ significantly between the Lake West treatments (Fig. 4b), similar to the ARG results. Procrustes (*M*^2^ = 0.33, *P* < 0.01, 9999 permutations) and Mantel (*r* = 0.46, *P* = 0.01) analyses found a strong correlation between the abundances of the ARGs and the bacterial communities for all samples (Fig. 4c). Specifically, the heatmap of the correlation between genera and ARG abundance indicated that the abundance of many genera were positively correlated with different ARG categories (Spearman, *R* > 0.6, *P* < 0.05) (Fig. 4d) and that the relative abundances of these genera were higher in the *Planktothrix* treatment group than other groups (Fig. 4e).

### Morphology of *Planktothrix* and *Microcystis* under scanning electron microscopy

*Planktothrix* was intertwined, creating a larger phycospheric space than *Microcystis* (Fig. S6). *Pseudanabaena*, another filamentous cyanobacterium belonging to the same order as *Planktothrix* (Oscillatoriales), dominated in all groups and provided a suitable phycospheric environment for abundant bacteria in our previous study, which focused on Lake Taihu^34^ (Fig. S6c). For quantification of cyanobacterial phycosphere surface area in detail, we managed to calculate the surface area of a single cyanobacterial cell and found that the surface area of *Planktothrix agardhii* (103–214.09 μm^2^, approximately) was larger than the surface area of *Microcystis aeruginosa* (28.26–153.86 μm^2^, approximately).

## Discussion

Based on the above results, we found remarkable interactions between the cyanobacterial bloom, the community composition of bacteria and the diversity and abundance of ARGs. *M. aeruginosa* blooms in the Xidong Reservoir affected the antibiotic resistome by affecting the aquatic microbial community^26^, and higher microbial activity promoted the spread of ARBs and ARGs in the eutrophic Lake Geneva^35,36^. Interestingly, in this study, two cyanobacteria (*Planktothrix* and *Microcystis*) occurred during the bloom and were associated with two completely different bacterial communities (Fig. S3). ARG composition differed observably between the *Planktothrix* bloom and the other sampling periods, indicating that *Planktothrix* blooms may indirectly affect ARG composition by affecting bacterial-community structure.

We therefore developed two hypotheses to account for this phenomenon: first, an abiotic factor that many ARBs may have been injected into the aquatic system from other habitats due to a series of adverse meteorological effects (such as heavy rain) during the *Planktothrix* bloom; second, a biotic factor caused by interactions between different cyanobacterial species and the ARB communities of Lake Taihu may have driven changes in ARG abundance and diversity.

To test these hypotheses, we checked the meteorological conditions during sampling and found that rainstorms were frequent during the *Planktothrix* bloom (Table S1). The 16S sequencing results also indicated that the composition of the main genera differed notably between the *Planktothrix* and *Microcystis* blooms. The abundance of Proteobacteria in the lake dramatically increased during the *Planktothrix* bloom, dominated by *Pseudomonas*, *Buttiauxella*, *Aeromonas* and *Flavobacterium*. Most of these genera are in the Pathogen-Host Interactions database (PHI-base) and are considered to be potential pathogens^37^, which may come from other habitats such as aerosols, soil, or human feces that were introduced due to rainfall. A recent study found that ARGs in the sediments of Lake Taihu were associated with human fecal contamination, which entered the lake via runoff during rainstorms^38,39^. These dominant genera are commonly resistant to antibiotics, especially to beta-lactams^40–42^, which could potentially account for the conspicuous increase in the abundance of beta-lactamase ARGs in the July samples.

Most importantly, the results also demonstrated the key role that MGEs have in determining ARG composition. MGEs are generally used by bacteria to store and express different exogenous resistance genes and play a crucial role in the dissemination of antibiotic resistance^43–45^. Various classes of ARGs are strongly correlated with mobile elements, which allow the transfer of ARGs between environmental bacteria in soil and freshwater^38,45^. We found a strong correlation between the MGEs and ARGs and a positive correlation between the main genera and MGEs, indicating the potential for the horizontal transfer of ARGs associated with MGEs.

The ratio of bacterial to fungal (B:F) richness was nine-fold higher during the bloom than the non-bloom period (Fig. S5a, b). A recent study used B:F as a parameter for microbial interactions and highlighted the importance of B:F in affecting ARG relative abundances^46^. Cyanobacterial-mediated interactions between bacteria and fungi may be a key factor in changes to ARG abundance during cyanobacterial blooms. We concluded that biotic processes (microbial interactions) could not be ignored in the succession of responses of ARG composition to cyanobacterial blooms. We thus designed a coculturing system to exclude abiotic factors for deciding whether cyanobacteria were an important factor determining ARG composition and the ARB community.

The characteristics of different species of cyanobacteria, such as morphology, type of toxin secreted or standing biomass, will generally help the development of different microbial communities^47^. The bacterial communities in laboratory microcosmic experiments differed greatly between the *Microcystis* and *Planktothrix* treatments, perhaps caused by species-specific allelochemicals^27^. This phenomenon has been demonstrated in comparisons between *Microcystis* and *Anabaena* blooms^47^. The ARG compositions in the *Microcystis* and *Planktothrix* treatments differed in both composition and diversity. The number and abundance of ARGs were dramatically higher in the *Planktothrix* treatments for both the urban river and Lake West water, similar to field results from Lake Taihu. Dias et al.^48^ recently reported that the sensitivities of *M. aeruginosa* and *P. agardhii* differed under different antibiotic pressures, with *P. agardhii* being naturally resistant to some antibiotics, allowing it to acquire resistance from the environment and to spread this resistance to other microorganisms^49^. The Pearson correlation that we calculated to determine the relationships between the cyanobacteria and ARGs and MGEs were nonetheless weakly correlated in the urban river and Lake West (Fig. S4).

Intrinsic or natural antibiotic resistance is a natural phenomenon that may be associated with 3% of the bacterial genome and is an important aspect of environmental resistance^50^. This resistance is not due to the horizontal transfer of ARGs, nor to the selective pressure of antibiotics, but to the inherent structural, physiological and biochemical properties of the bacterial cells^50,51^. We also maintained a low-density addition of cyanobacteria in the coculturing system to rule out the effects of cyanobacteria. We could therefore ensure that the assembly of resistant communities in the biotic processes were mainly due to variations in the bacterial community, which were affected by the allelopathic effect of the cyanobacteria rather than by the direct impact of cyanobacteria containing ARGs.

Interestingly, our results suggested that aquatic bacteria and *Planktothrix* could be symbiotic mutualists, which may increase the probability of ARBs. Cyanobacteria typically release various low-molecular-weight molecules during blooms, including glucose, organic acids, amino acids and sugar alcohols, which may benefit phycospheric bacteria^7,52^. One study has demonstrated that *Planktothrix* could resist parasitic fungi by secreting hassallidin, an antifungal peptide^53^. We found in a previous study that an antifungal agent (azoxystrobin) could enrich ARGs in the gut of *Enchytraeus crypticus*^29^.

ARG numbers in the urban river and Lake West microcosms were slightly lower in the *Microcystis* treatment, which paralleled the results from Lake Taihu, due to the secretion of toxic substances by *M. aeruginosa*, which could affect the associated ARB community. A previous study found that *M. aeruginosa* inhibited some key pathways of energy metabolism (glycolysis, TCA cycle and sugar metabolism) in aquatic microorganisms by producing a variety of toxic substances^34^. A recent study by Guo et al.^26^ also found that the abundances and the detected numbers of ARGs decreased considerably during a bloom of *M. aeruginosa*.

We found that many of the abundant genera responded similarly to the different cyanobacteria within the two natural aquatic environments, demonstrating that *Planktothrix* and *Microcystis* specifically affected the surrounding microbial communities. The abundances of these genera were also strongly correlated with ARG abundance, and the genera were abundant in the *Planktothrix* treatment. For example, *Bacteroidetes* was more abundant in the *Planktothrix* than the *Microcystis* treatment. The correlation analyses demonstrated that the abundances of *Saprospiraceae*, *Haliscomenobacter* and the NS11-12 marine group, all of which are Bacteroidetes, were all strongly correlated with ARG abundance (Pearson, *R* > 0.6, *P* < 0.01). Other studies have also reported that Bacteroidetes abundance was strongly correlated with ARG abundance in the animal gut^54^, soil^55^, and wastewater^56^, which supports our conclusion that *Planktothrix* contributes to the growth and emergence of ARBs by influencing the growth of Bacteroidetes.

According to the morphology of *Planktothrix* and *Microcystis* under scanning electron microscopy, this filamentous cyanobacterium can provide a larger habitat for the survival of bacteria, which is termed as the “cyanosphere”^7^: the region in the immediate vicinity of cyanobacteria containing rich cyanobacterial secretions. Moreover, compared with other filamentous cyanobacteria, *Planktothrix* has a stronger carbon and nitrogen metabolism capacity for the use of surrounding bacteria^57^. These beneficial commensal bacteria may be part of the ARB community. Bacterial communities mediated by cyanobacterial blooms may have different ecological functions, which could be an important perspective to understand the bacteria that contribute to the appearance and spread of ARGs. We used the Tax4Fun method to predict aquatic bacterial functions and annotated them at KEGG (Kyoto Encyclopedia of Genes and Genomes) level 3. This method identified a noteworthy increase in the synthesis and metabolic pathways of carbohydrates and some essential amino acids after coculturing with *P. agardhii* compared to the *Microcystis* treatment (Fig. S7a), indicating that the prosperous growth of bacterial communities could be mediated by *Planktothrix*. The pressure of environmental antibiotics may also be an important factor in the emergence and spread of ARGs^58,59^. We therefore also examined changes to the pathways for the biosynthesis of some common antibiotics (Fig. S7b) but found no obvious difference between the control and cocultured groups.

All these results suggest that *Planktothrix* increases the probability of emergence of ARBs by increasing bacterial populations in the phycosphere and by secreting nutrients that are beneficial to bacterial growth. *Planktothrix* also secreted some antifungal substances to promote the emergence and spread of ARGs.

In conclusion, the ARB communities had clearly different patterns of emergence during the different phases of cyanobacterial blooms, and both biotic and abiotic processes contributed to these changes in community structure. Our results demonstrated that changes in ARG abundance were mainly due to changes in the composition and structure of the ARB communities. The structure of the ARB community throughout the cyanobacterial bloom was more stable than the structure of the entire bacterial community. Our results also indicated that the cyanobacterial blooms increased the interactions between microorganisms and affected the composition of resistant communities.

Our laboratory results also suggested that the same phenomenon can occur in other environments (urban river and Lake West) and that cyanobacteria affected the ARB communities by secreting specific chemicals. *Planktothrix* was potentially symbiotic with ARBs and could secrete antifungal substances to promote the emergence and spread of the ARBs. Our study found that cyanobacterial blooms, a global ecological problem, could be a key driver of ARG diffusion and enrichment in freshwater. More critically, since many *Planktothrix* blooms occur in nearshore embayment and river mouths close to human populations, there are potential broad implications for human health impacts that extend beyond the typical cyanotoxin threats. Our results are useful for identifying the ecological risks in global freshwater lakes and provide a reference for assessing and managing water quality.

## Methods and materials

### Sample acquisition and field data

Samples were collected from Meiliang Bay, the main location of cyanobacterial blooms in northern Lake Taihu as our previous study described^28^. Samples were collected during five periods (July, August and September 2016 and March and May 2017) at three locations (A: 31°31′19″N, 120°13′49″E; B: 31°32′01″N, 120°13′15″E; C: 31°32′31″N, 120°13′33″E). About 12 L of lake water were collected at a depth of 0.5 m at each location, the water temperature was measured directly, and pH was measured in the laboratory using a pH meter (FE20, Mettler Toledo, Zurich, Switzerland). Each sample was filtered through 0.22-µm polycarbonate filters to obtain all aquatic microorganisms, which were frozen at −80 °C. The concentrations of total nitrogen (TN), total phosphorus (TP), nitrate (NO_3_^−^) and ammonium (NH_4_^+^) were measured in the filtered water following the methods described our previous studies^28^.

### DNA extraction from aquatic microorganisms for high-throughput sequencing

Filtered samples were prepared for DNA extraction using the Power Soil DNA Isolation Kit (Biomiga, Carlsbad, USA). The quality of the isolated DNA was assessed by 0.8% agarose gel electrophoresis, and the concentration was measured spectrophotometrically (Nanodrop ND-1000, Thermo Fisher NanoDrop, Wilmington, Delaware, USA). We selected the primer pair 341F (CCTAYGGGRBGCASCAG) and 806R (GGACTACNNGGGTATCTAAT) to target the V3-V4 hypervariable region of the 16S rRNA gene (one of the least overestimates regions^60^), with unique barcodes on the reverse primer. The primers ITS5-1737F (GGAAGTAAAAGTCGTAACAAGG) and ITS2-2043R (GCTGCGTTCTTCATCGATGC) were used to amplify the ITS rRNA gene on the Illumina HiSeq2500 platform (Illumina, San Diego, California, USA). The Phusion® High-Fidelity PCR Master Mix (New England Biolabs, Hertfordshire, UK) was used for all PCRs.

### Real-time quantitative PCR (qPCR)

The absolute number of 16S genes in the samples was quantified using Applied Biosystems StepOneTM and StepOnePlusTM Real-Time PCR Systems (Thermo Fisher Scientific, Waltham, USA) with SYBR® Green. The 20-μL qPCR reactions contained 10 μL of 2× buffer (Master mix, TOYOBO, Osaka, Japan), 0.8 μL of each primer, 4.4 μL of sterile distilled water and 4 μL of template DNA (30 μg μL^−1^). The qPCR amplification for each sample was run in triplicate as described by Ouyang et al.^61^: pre-incubation at 95 °C for 5 min followed by 40 cycles of 95 °C for 15 s, 60 °C for 1 min and 72 °C for 15 s. The standard curve used to quantify the absolute number of 16S genes was generated using the method described by Qian et al.^62^: log (16S gene number) = −0.3664 × Ct + 14.405 (*R*^2^ = 0.9955).

### Analysis of ARGs by HT-qPCR

HT-qPCR was performed using the SmartChip Real-Time PCR System (Warfergen Inc., Fremont, USA) to investigate the relative abundance and composition of the ARGs. We selected 296 primer pairs that targeting one 16S gene, 285 major ARGs and 10 MGEs (1 clinical class 1 integron, 1 class 1 integron and eight transposases). The HT-qPCR protocol has been described by Zhang et al.^29^, and SmartChip qPCR software was used to analyze the data. All samples were tested with three technical replicates, and Ct was set to 31. We obtained the ARG abundance per cell using normalized numbers of the 16S gene, as in previous studies^30,32^. The absolute numbers of ARGs were calculated by normalization to absolute 16S gene numbers^26^.

### Laboratory coculture experimental verification of the relationship between cyanobacteria and the ARG resistome

Surface water from a depth of 0.5 m was sampled from Lake West and an urban river in Hangzhou (Zhejiang, China) to obtain samples of plankton (Fig. S1). Physicochemical parameters of the samples, i.e., pH and TN, TP, NO_3_^−^, and NH_4_^+^ concentrations, were measured as described above. The common cyanobacterial species *Planktothrix agardhii* and *Microcystis aeruginosa* were purchased from the Institute of Hydrobiology, Chinese Academy of Sciences (Wuhan, China). We verified the effects of *Microcystis* and *Planktothrix* on the ARB community and ARG abundance by adding 2% (g/g, calculated using the biomasses of the cyanobacterial and plankton samples) *P. agardhii* and *M. aeruginosa* to 150 mL of the plankton samples in 250-mL flasks. The plankton was cultured under a cool-white fluorescence light at 46 μmol m^−2^ s^−1^ with a 12/12 h light/dark cycle at 25 ± 0.5 °C. All flasks were shaken gently three times a day to mix the cyanobacteria and plankton. The experimental groups setting was divided into two classes, an urban river: UC, UM and UP; West Lake: WC, WM and WP respectively represent controls, co-cultured with *M. aeruginosa*, and co-cultured with *P. agardhii*. Cultures were prepared in quadruplicate for each treatment.

Samples of 150 mL from each group were filtered through 0.22-µm polycarbonate filters after 7 d of culture to recover all microorganisms for DNA extraction. High-throughput sequencing and HT-qPCR were then used for the analysis of the ARB community and ARG abundance as described above. New primer pairs, 338F (ACTCCTACGGGAGGCAGCAG) and 806R (GGACTACHVGGGTWTCTAAT), were used in the experiments to target the V3-V4 hypervariable region of the 16S gene.

### Observation of cyanobacterial morphology under scanning electron microscopy

The *M. aeruginosa*, *P. agardhii* and the Lake Taihu microcosmic samples were collected by centrifugation (3000 rpm, 15 min) after seven days of culturing and fixed with 2.5% glutaraldehyde at 4 °C overnight. Each sample was then washed for 15 min in phosphate buffer solution (0.1 M, pH 7.0) and postfixed with 1% OsO_4_ in phosphate buffer for 1–2 h before washing several times with phosphate buffer for 15 min. The samples were next dehydrated using increasing concentrations of ethanol (30, 50, 70, 80, 90, and 100%) for ~15 min for each step. The samples in the last step were coated with gold-palladium and observed with a Hitachi Model TM-1000 SEM^27^.

### Statistics and reproducibility

We used a one-way ANOVA with a post hoc test (Fisher’s protected least significant difference) using StatView 5.0 to identify significant differences (*P* < 0.05). Procrustes analyses and Mantel tests were used to analyze the correlation between the ARG and OTU data (bacterial and fungal) in R version 3.4.1 with vegan 2.4−3. We conducted a redundancy analysis (RDA) and a partial redundancy analysis (pRDA) in the vegan 2.3−1 package and calculated the correlations between the ARG and genus data using corrplot version 0.84. Co-occurrence network graphs were visualized using Cytoscape 3.6.0, with the strong correlations (Spearman’s *r* > 0.7, *P* < 0.01) identified using the psych package in R3.5.1.

A principal coordinate analysis (PCoA) of the ARGs was generated using Origin 2018, and its dimension-reduction coordinates were calculated using the vegan 2.3-1 package in RStudio. A heatmap of the correlations between the ARGs and genera was produced using the Meiji Cloud Biological Platform. Bray–Curtis similarity was used to identify the ARGs and OTUs contributing most to the dissimilarity between months, and graphs were generated using GraphPad Prism 7.00. All other histograms and pie and line charts were produced using GraphPad Prism 7.00. Tax4Fun, an open-source R package based on the SILVA taxonomy and the Kyoto Encyclopedia of Genes and Genomes (KEGG) databases, was used to predict the functional attributes of the bacterial communities in our laboratory experiments.

### Reporting summary

Further information on research design is available in the Nature Research Reporting Summary linked to this article.

## Supplementary information

Supplementary Information

Reporting Summary

Description of Additional Supplementary Files

Supplementary Data 1

Supplementary Data 2

Supplementary Data 3

Supplementary Data 4

## Data Availability

The original raw sequencing data have been published in the NCBI Sequence Read Archive (SRA) database with the BioProject numbers PRJNA513111 (raw data of 16S in field work), PRJNA517290 (raw data of ITS in field work) and PRJNA658773 (raw data of 16S in laboratory coculture experiment). Source data underlying plots shown in figures are provided in Supplementary Data 1–3. Any other data, if any, can be provided upon request by the corresponding author.

## Code availability

The R codes used for the Procrustes and mantel test and Redundancy analysis in this study are available online at https://github.com/ZhenyanZhang/Communications-biology-Zhang-2020.
