# Supplementary material for: Cyanobacterial blooms contribute to the diversity of antibiotic-resistance genes in aquatic ecosystems
Source: Commun Biol. 2020 Dec 4;3:737. doi: 10.1038/s42003-020-01468-1 (PMC7718256; doi:10.1038/s42003-020-01468-1)
Supplement: Supplementary file 1 — Supplementary Information [file 42003_2020_1468_MOESM1_ESM.pdf]

Supplementary Information

Cyanobacterial blooms contribute to the diversity of antibiotic-resistance genes in aquatic ecosystems

Qi Zhang[1#], Zhenyan Zhang[1#], Tao Lu[1], W.J.G.M. Peijnenburg[2, 3], Michael Gillings[4], Xiaoru Yang[5], Jianmeng Chen[1], Josep Penuelas[6,7], Yong-Guan Zhu[5,8], Ning-Yi Zhou[9], Jianqiang Su[5, *], Haifeng Qian[1, *]

1. College of Environment, Zhejiang University of Technology, Hangzhou 310032, P. R. of China;

2. Institute of Environmental Sciences (CML), Leiden University, 2300 RA Leiden, The Netherlands;

3. National Institute of Public Health and the Environment (RIVM), Center for Safety of Substances and Products, P.O. Box 1, Bilthoven, The Netherlands;

4. Department of Biological Sciences, Macquarie University, Sydney, NSW 2109, Australia;

5. Key Laboratory of Urban Environment and Health, Institute of Urban Environment, Chinese Academy of Sciences, Xiamen 361021, P. R. of China;

6. CSIC, Global Ecology Unit CREAF-CSIC-UAB, Bellaterra, 08193 Barcelona, Catalonia, Spain;

7. CREAF, Cerdanyola del Vallès, 08193 Barcelona, Catalonia, Spain;

8. State Key Lab of Urban and Regional Ecology, Research Center for Ecoenvironmental Sciences, Chinese Academy of Sciences, Beijing 100085, P. R. of China

9. State Key Laboratory of Microbial Metabolism, and School of Life Sciences & Biotechnology, Shanghai Jiao Tong University, Shanghai, 200240, P. R. of China

---

[#] Qi Zhang and Zhenyan Zhang contributed equally to this work.

[*]To whom correspondence should be addressed. Email: jqsu@iue.ac.cn (J.Q.S.), hfqian@zjut.edu.cn (H.F.Q.).

Supplementary Table S1. The weather condition of July and August 2016.

Supplementary Table S2. Total primers of ARG.

Supplementary Figure S1. The changes of physicochemical water parameters (temperature, pH, TN, TP, NO_3^- and NH_4^+) of Lake Taihu across spatial and temporal scales (a). *Planktothrix* (*CN-CO*) and *Microcystis* (*CH-CI*) specific gene expression determined by qRT-PCR (b). Correlations between ARGs and bacterial (c) or fungal (d) communities were determined by Procrustes and Mantel analysis. Principal coordinate analysis (PCoA) of ARGs communities in Lake Taihu using weighted Unifrac distances (e).

Supplementary Figure S2. The relative abundance of bacterial communities at phylum (>2%) in Urban River (UC) and West Lake (WC) co-cultured with the *Planktothrix agardhii* (UP and WP) and *Microcystis aeruginosa* (UM and WM) after 7 days (mean ± SE, n = 4).

Supplementary Figure S3. Principal coordinate analysis (PCoA) of Lake Taihu bacteria samples of different months using weighted Unifrac distances.

Supplementary Figure S4. Heatmap describing the correlation between phylum level (Top 3) and different ARG categories in the unban river and West Lake co-culture systems. “**” and “***” indicate statistically significant differences between the bacterial and ARG communities ($P<0.01$ and $P<0.001$, one-way ANOVA).

Supplementary Figure S5. Partial redundancy analysis differentiating the effects of bacterial richness/fungal richness (B:F) and environmental factors (EF) on the variations in ARG abundance during the non-bloom (a) and bloom (b) stages.

Supplementary Figure S6. Aquatic microbiota functions in the unban river and West Lake predicted by the Tax4Fun method. KEGG level 3 inferred functional annotations that differed by LEfSe analysis (a). The relative abundances of pathways related with antibiotic biosynthesis (b).

Supplementary Figure S7. Scanning electron microscope (SEM) images of the *Planktothrix agardhii* (1000×) (a) and *Microcystis aeruginosa* after (3000×) after 7 days cultured in BG11 medium (b); The Lake Taihu sample cultured in BG11 medium for 7days (c)

JULY 2016

一	二	三	四	五	六	日
						1 Heavy rain
2 Rainstorm	3 Rainstorm	4 Rainstorm	5 Moderate rain	6 Moderate rain	7 Light rain	8 Light rain
9 Light rain	10 Light rain	11 Light rain	12 Light rain	13 Light rain	14 Moderate rain	15 Moderate rain
16 sampling	17	18	19	20	21	22
23	24	25	26	27	28	29
30	31					

August 2016

一	二	三	四	五	六	日
						1 Thundershower
2 Thundershower	3 Moderate rain	4 Cloudy	5 Cloudy	6 Cloudy	7 Showers	8 Moderate rain
9 Thundershower	10 Moderate rain	11 Rainstorm	12 Thundershower	13 Thundershower	14 Cloudy	15 Cloudy
16 sampling	17	18	19	20	21	22
23	24	25	26	27	28	29

30	31					

Supplementary Table S1. The weather condition of July and August 2016.

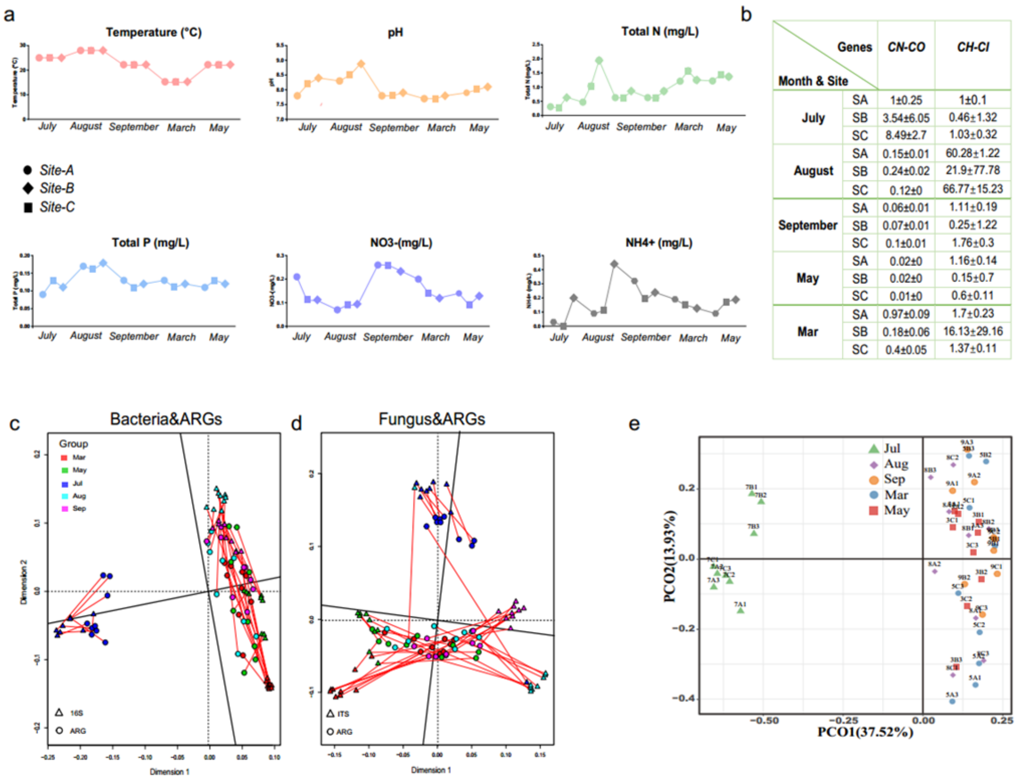

Month & Site		CN-CO	CH-CI
July	SA	1±0.25	1±0.1
	SB	3.54±6.05	0.46±1.32
	SC	8.49±2.7	1.03±0.32
August	SA	0.15±0.01	60.28±1.22
	SB	0.24±0.02	21.9±77.78
	SC	0.12±0	66.77±15.23
September	SA	0.06±0.01	1.11±0.19
	SB	0.07±0.01	0.25±1.22
	SC	0.1±0.01	1.76±0.3
May	SA	0.02±0	1.16±0.14
	SB	0.02±0	0.15±0.7
	SC	0.01±0	0.6±0.11
Mar	SA	0.97±0.09	1.7±0.23
	SB	0.18±0.06	16.13±29.16
	SC	0.4±0.05	1.37±0.11

Supplementary Figure S1. The changes of physicochemical water parameters (temperature, pH, TN, TP, NO3- and NH4+) of Lake Taihu across spatial and temporal scales (a). Planktothrix (CN-CO) and Microcystis (CH-CI) specific gene expression determined by qRT-PCR (b). Correlations between ARGs and bacterial (c) or fungal (d) communities were determined by Procrustes and Mantel analysis. Principal coordinate analysis (PCoA) of ARGs communities in Lake Taihu using weighted Unifrac distances (e).

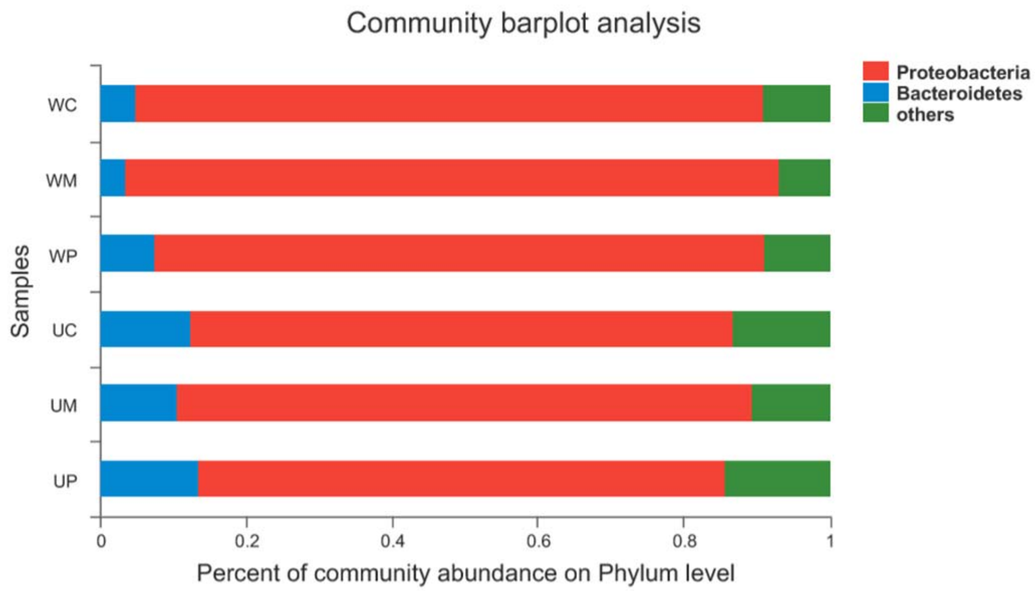

Supplementary Figure S2. The relative abundance of bacterial communities at phylum (>2%) in Urban River (UC) and West Lake (WC) co-cultured with the *Planktothrix agardhii* (UP and WP) and *Microcystis aeruginosa* (UM and WM) after 7 days (mean ± SE, n = 4).

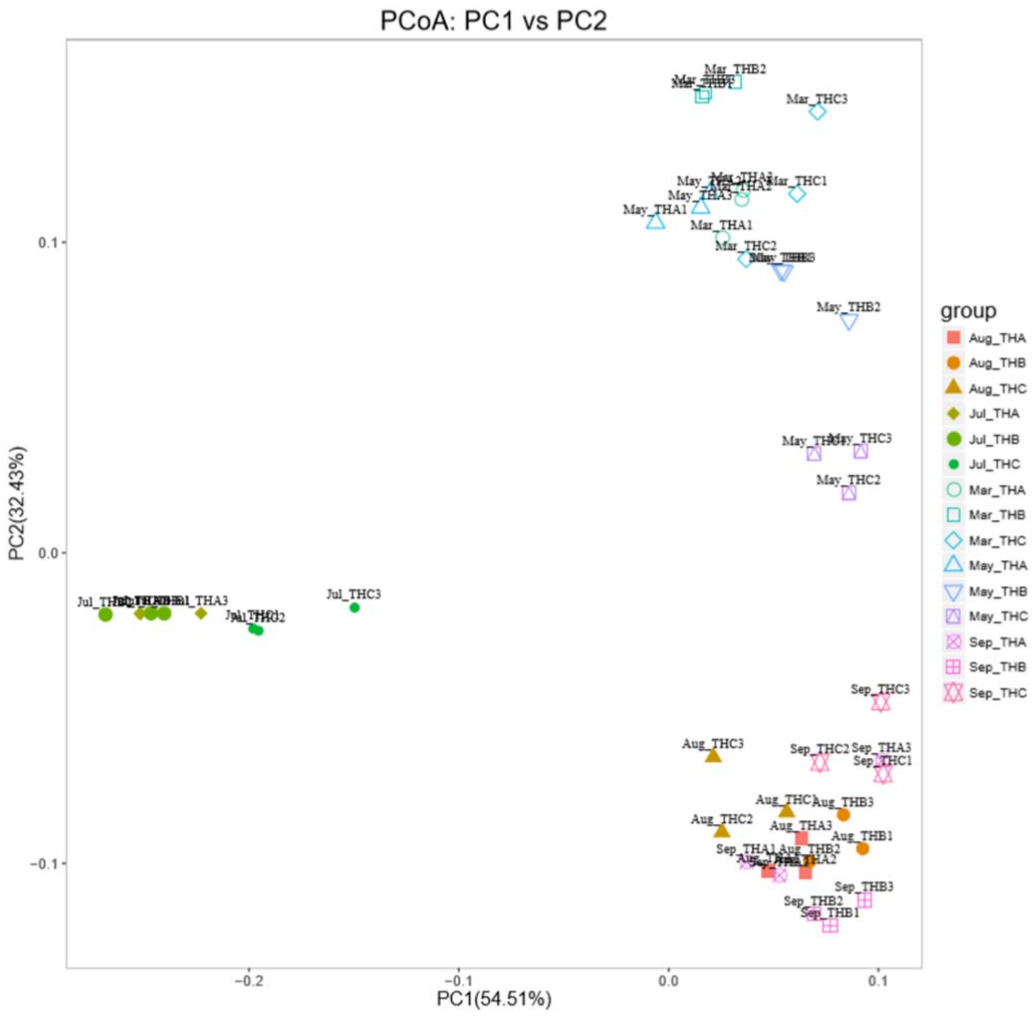

Supplementary Figure S3. Principal coordinate analysis (PCoA) of Lake Taihu bacteria samples of different months using weighted Unifrac distances.

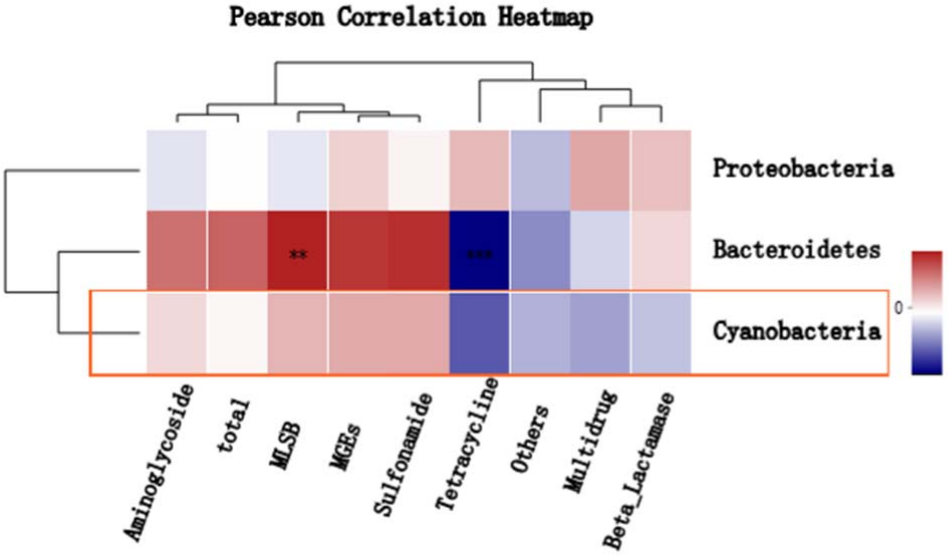

Supplementary Figure S4. Heatmap describing the correlation between phylum level (Top 3) and different ARG categories in the unban river and West Lake co-culture systems. “**” and “***” indicate statistically significant differences between the bacterial and ARG communities (P<0.01 and P<0.001, one-way ANOVA).

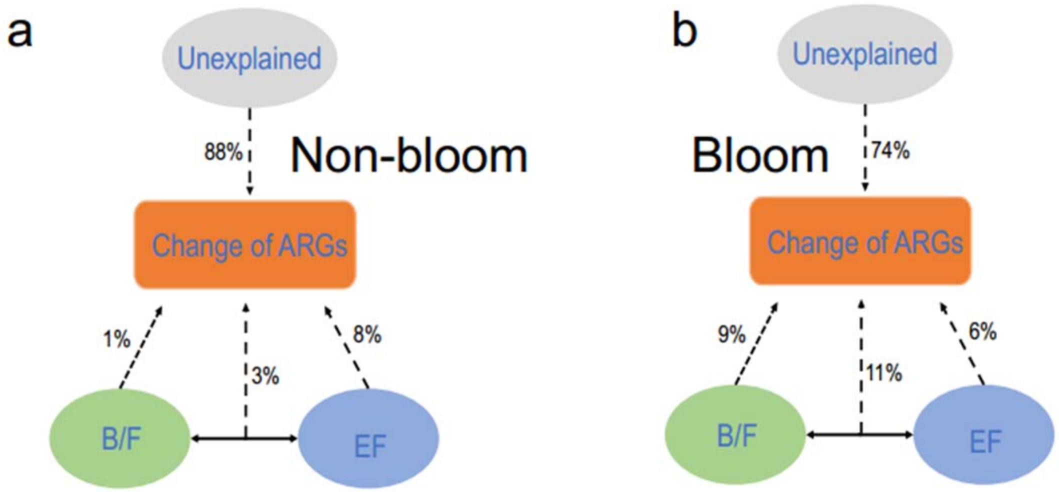

Supplementary Figure S5. Partial redundancy analysis differentiating the effects of bacterial richness/fungal richness (B:F) and environmental factors (EF) on the variations in ARG abundance during the non-bloom (a) and bloom (b) stages.

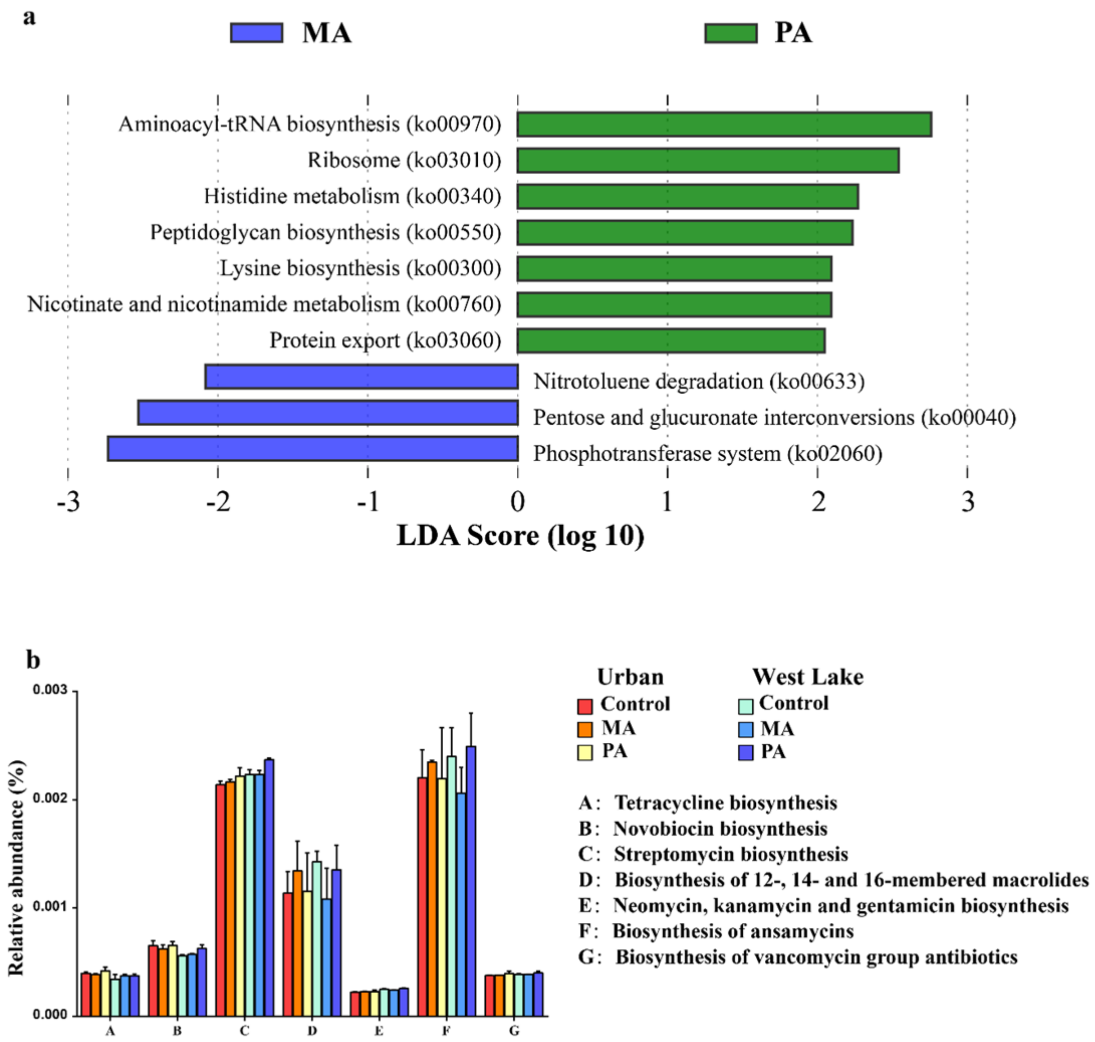

Supplementary Figure S6. Aquatic microbiota functions in the unban river and West Lake predicted by the Tax4Fun method. KEGG level 3 inferred functional annotations that differed by LEfSe analysis (a). The relative abundances of pathways related with antibiotic biosynthesis (b).

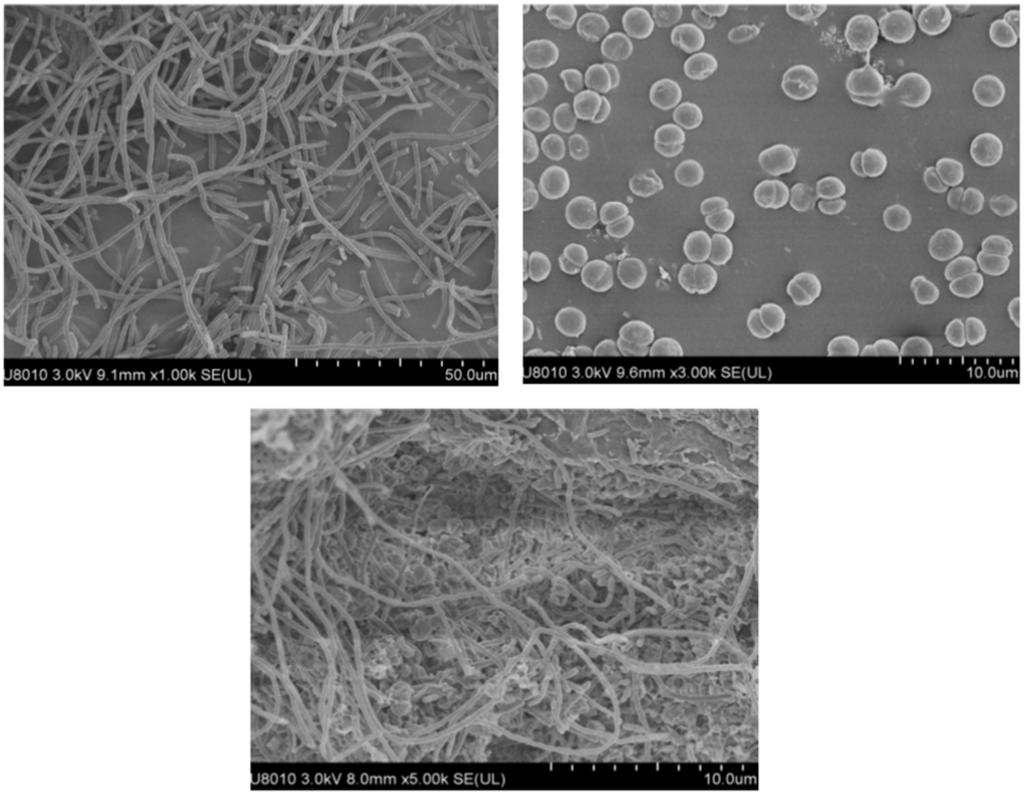

Supplementary Figure S7. Scanning electron microscope (SEM) images of the *Planktothrix agardhii* (1000×) (a) and *Microcystis aeruginosa* after (3000×) after 7 days cultured in BG11 medium (b); The Lake Taihu sample cultured in BG11 medium for 7days (c).
