# Supplementary material for: Cyanobacterial blooms contribute to the diversity of antibiotic-resistance genes in aquatic ecosystems
Source: Commun Biol. 2020 Dec 4;3:737. doi: 10.1038/s42003-020-01468-1 (PMC7718256; doi:10.1038/s42003-020-01468-1)
Supplement: Supplementary file 2 — Reporting Summary [file 42003_2020_1468_MOESM2_ESM.pdf]

nature research

Corresponding author(s): Haifeng Qian

Last updated by author(s): Aug 24, 2020

Reporting Summary

Nature Research wishes to improve the reproducibility of the work that we publish. This form provides structure for consistency and transparency in reporting. For further information on Nature Research policies, see Authors & Referees and the Editorial Policy Checklist .

Statistics

For all statistical analyses, confirm that the following items are present in the figure legend, table legend, main text, or Methods section.

n/a	Confirmed	
☐	☒	The exact sample size (*n*) for each experimental group/condition, given as a discrete number and unit of measurement
☐	☒	A statement on whether measurements were taken from distinct samples or whether the same sample was measured repeatedly
☐	☒	The statistical test(s) used AND whether they are one- or two-sided<br>*Only common tests should be described solely by name; describe more complex techniques in the Methods section.*
☒	☐	A description of all covariates tested
☒	☐	A description of any assumptions or corrections, such as tests of normality and adjustment for multiple comparisons
☐	☒	A full description of the statistical parameters including central tendency (e.g. means) or other basic estimates (e.g. regression coefficient) AND variation (e.g. standard deviation) or associated estimates of uncertainty (e.g. confidence intervals)
☐	☒	For null hypothesis testing, the test statistic (e.g. *F*, *t*, *r*) with confidence intervals, effect sizes, degrees of freedom and *P* value noted<br>*Give P values as exact values whenever suitable.*
☒	☐	For Bayesian analysis, information on the choice of priors and Markov chain Monte Carlo settings
☐	☒	For hierarchical and complex designs, identification of the appropriate level for tests and full reporting of outcomes
☐	☒	Estimates of effect sizes (e.g. Cohen's *d*, Pearson's *r*), indicating how they were calculated

Our web collection on statistics for biologists contains articles on many of the points above.

Software and code

Policy information about availability of computer code

Data collection	For gaining rawdata of 16s and 18s rRNA (Flash 1.2.11; Qiime 1.9.1; Fastp 0.19.6;); the antibiotic resistance genes data (SmartChip qPCR); the absolute number of 16S genes (StepOne Software v2.3); the scanning electron microscopy imagin (Image-Pro Plus software).
Data analysis	We used a one-way ANOVA with a post hoc test (Fisher's protected least significant difference) using StatView 5.0 to identify significant differences (P<0.05). Procrustes analyses and Mantel tests were used to analyze the correlation between the ARG and OTU data (bacterial and fungal) in R version 3.4.1 with vegan 2.4−3. We conducted a redundancy analysis (RDA) and a partial redundancy analysis (pRDA) in the vegan 2.3−1 package and calculated the correlations between the ARG and genus data using corrplot version 0.84. Co-occurrence network graphs were visualized using Cytoscape 3.6.0, with the strong correlations (Spearman's r>0.7, P<0.01) identified using the psych package in R3.5.1.<br>A principal coordinate analysis (PCoA) of the ARGs was generated using Origin 2018, and its dimension-reduction coordinates were calculated using the vegan 2.3−1 package in RStudio. A heatmap of the correlations between the ARGs and genera was produced using the Meiji Cloud Biological Platform. Bray−Curtis similarity was used to identify the ARGs and OTUs contributing most to the dissimilarity between months, and graphs were generated using GraphPad Prism 7.00. All other histograms and pie and line charts were produced using GraphPad Prism 7.00. Tax4Fun, an open-source R package based on the SILVA taxonomy and the Kyoto Encyclopedia of Genes and Genomes (KEGG) databases, was used to predict the functional attributes of the bacterial communities in our laboratory experiments.

For manuscripts utilizing custom algorithms or software that are central to the research but not yet described in published literature, software must be made available to editors/reviewers. We strongly encourage code deposition in a community repository (e.g. GitHub). See the Nature Research guidelines for submitting code & software for further information.

Data

Policy information about availability of data

All manuscripts must include a data availability statement. This statement should provide the following information, where applicable:

- Accession codes, unique identifiers, or web links for publicly available datasets
- A list of figures that have associated raw data
- A description of any restrictions on data availability

The original raw sequencing data have been published in the NCBI Sequence Read Archive (SRA) database with the BioProject numbers PRJNA513111 (raw data of 16S in field work), PRJNA517290 (raw data of ITS in field work) and PRJNA658773 (raw data of 16S in laboratory coculture experiment)

Field-specific reporting

Please select the one below that is the best fit for your research. If you are not sure, read the appropriate sections before making your selection.

☐ Life sciences ☐ Behavioural & social sciences ☒ Ecological, evolutionary & environmental sciences

For a reference copy of the document with all sections, see nature.com/documents/nr-reporting-summary-flat.pdf

Ecological, evolutionary & environmental sciences study design

All studies must disclose on these points even when the disclosure is negative.

Study description	We firstly investigated the effect of cyanobacterial blooms on ARG composition by a field study in Lake Taihu, which suffered from cyanobacterial blooms annually. Then, to confirm the results of field study in other aquatic habitats, we carried out a microcosm experiment using the freshwater collected from the Lake West and a typical urban river. The common cyanobacterial species Planktothrix agardhii and Microcystis aeruginosa, which dominated in July and August respectively as the field study showed, were added into the microcosm and cultured for 7 days. The composition of bacterial communities and ARGs were determined by 16S rRNA gene sequencing and HT-qPCR.
Research sample	The total DNA of water samples collected from the field study and the microcosm experiment. In the field study, water samples were collected from Meiliang Bay, the main location of cyanobacterial blooms in northern Lake Taihu, during five periods (July, August and September 2016 and March and May 2017) at three locations (A: 31°31′19″N, 120°13′49″E; B: 31°32′01″N, 120°13′15″E; C: 31°32′31″ N, 120°13′33″E). For microcosm experiment, the experimental groups setting was divided into two classes, an urban river: UC, UM and UP; West Lake: WC, WM and WP respectively represent controls, co-cultured with M. aeruginosa, and co-cultured with P. agardhii. After 7 days culture, water samples were collected in quadruplicate in each group.
Sampling strategy	For the field study: water samples were collected from three locations of Meiliang Bay, the main location of cyanobacterial blooms in northern Lake Taihu, during non-bloom months (March and May 2017) and bloom months (July, August and September 2016). Samples were collected in triplicate in each group is enough for determining the composition of bacterial communities and ARGs.<br>For microcosm experiment: Lake West and the urban river in Hangzhou, China were chosen as the representative of the urban lake and river environment. The common cyanobacterial species Microcystis aeruginosa and Planktothrix agardhiiwere chosen as they were dominated in July and August respectively, as the field study showed. Samples were collected in quadruplicate in each group is enough for determining the composition of bacterial communities and ARGs.
Data collection	We sent the DNA samples obtained from the filed and laboratory experiments to the sequencing company for sequencing. The obtained rawdata was analyzed and plotted by by the co-authors Qi zhang and Zhenyan Zhang. In addition, the physical and chemical properties and composition of the water body, algae growth counts, and algae morphology measurement data were recorded and analyzed by the co-author Qi zhang and Zhenyan Zhang.
Timing and spatial scale	In the field experiment, we took samples from July, August, September, 2016, and March 16, May 2017 (adverse weather extension) according to the early, middle, and late stages of the bloom period. For indoor experiments, we took water samples from the urban rivers and Lake West communities of Hangzhou and co-cultivated the monoalgae (Microcystis aeruginosa and Planktothrix agardhii), whcih were cultivated in our laboratory for several years, and collected samples on the seventh day.
Data exclusions	We declared that the experimental data of this manuscript were not excluted from analysis.
Reproducibility	We strictly control the standardization of every step of the operation during the experiment. For example, inoculation of all indoor experiments is carried out under sterile conditions, and the pure cyanobacteria are tested under the microscope after they are determined to be pollution-free. The time and place of field sampling are strictly controlled at the same time and at the same place.
Randomization	We conducted a year-long monitoring of areas with frequent blooms in LakeTaihu to identify two blooms of cyanobacteria (Microcystis and Planktothrix) and then built a microcosm (typical urban rivers and lakes) to co-culture with the two cyanobacteria. The entire experiment is deterministic.
Blinding	The blinding test is not applicable to our experiments, because all of our experimental data are obtained objectively, and are uniformly obtained through instrument determination, and there is no subjective factor.

Did the study involve field work? [x] Yes [ ] No

Field work, collection and transport

Field conditions	16 July, 2016: Cloudy, 24-29 ℃; 16 August, 2016: Cloudy, 27-36 ℃; 18 September, 2016: Cloudy, 21-27 ℃; 16 March,2017: Cloudy, 5-13 ℃; 16 May, 2017: Cloudy, 15-26 ℃.
Location	Three locations in Mayliang Bay (A: 31°31′19″N, 120°13′49″E; B: 31°32′01″N, 120°13′15″E; C: 31°32′31″N, 120°13′33″E)
Access and import/export	not applicable
Disturbance	We accurately located each sampling point through Google satellite map and chose the sunny or cloudy weather for sampling.

Reporting for specific materials, systems and methods

We require information from authors about some types of materials, experimental systems and methods used in many studies. Here, indicate whether each material, system or method listed is relevant to your study. If you are not sure if a list item applies to your research, read the appropriate section before selecting a response.

Materials & experimental systems

n/a	Involved in the study	
[x]	[ ]	Antibodies
[x]	[ ]	Eukaryotic cell lines
[x]	[ ]	Palaeontology
[x]	[ ]	Animals and other organisms
[x]	[ ]	Human research participants
[x]	[ ]	Clinical data

Methods

n/a	Involved in the study	
[ ]	[x]	ChIP-seq
[x]	[ ]	Flow cytometry
[x]	[ ]	MRI-based neuroimaging

ChIP-seq

Data deposition

[x] Confirm that both raw and final processed data have been deposited in a public database such as GEO.

[x] Confirm that you have deposited or provided access to graph files (e.g. BED files) for the called peaks.

Data access links<br>*May remain private before publication.*	https://www.ncbi.nlm.nih.gov/bioproject/PRJNA513111<br>https://www.ncbi.nlm.nih.gov/bioproject/PRJNA517290<br>https://www.ncbi.nlm.nih.gov/bioproject/PRJNA658773
Files in database submission	SRR8398881 to SRR8398925 (16S) and SRR8491695 to SRR8491739 (ITS)
Genome browser session<br>(e.g. UCSC)	not applicable

Methodology

Replicates	Samples were collected in triplicate in each group is enough for determining the composition of bacterial communities and ARGs and Samples were collected in quadruplicate in each group is enough for determining the composition of bacterial communities and ARGs in lab study.
Sequencing depth	In fied test, totals of 1 957 979 (bacterial) and 1 643 396 (fungal) high-quality sequences were obtained by assembling and quality filtering, with 27 593-60 812 bacterial samples and 28 499-46 939 fungal samples sequenced. In lab test, a total of 1 121 479 sequences were obtained from the 24 samples.
Antibodies	not applicable
Peak calling parameters	not applicable
Data quality	Uparse software (Uparse V 7.0.1090, http://www.drive5.com/uparse/) was used for sequence analysis after the filtration of the raw tags by Qiime (V1.9.1, http://qiime.org/install/index.html).
Software	Flash 1.2.11; Qiime 1.9.1; Fastp 0.19.6;
