# Supplementary material for: Cyanobacterial blooms contribute to the diversity of antibiotic-resistance genes in aquatic ecosystems
Source: Commun Biol. 2020 Dec 4;3:737. doi: 10.1038/s42003-020-01468-1 (PMC7718256; doi:10.1038/s42003-020-01468-1)
Supplement: Supplementary file 3 — Description of Additional Supplementary Files [file 42003_2020_1468_MOESM3_ESM.pdf]

Description of Additional Supplementary Files

File Name: Supplementary Data 1
Description: Figure 1d, 2 source data.
The relative abundance of the detected ARGs during the cyanobacterial blooms in Lake Taihu at site A, B and C.

File Name: Supplementary Data 2
Description: Figure 3 source data.
The relative abundance of the detected ARGs from the different co-culture systems, including the urban river and Lake West cocultured with *Microcystis aeruginosa* (+Ma) and *Planktothrix agardhii* (+Pa).

File Name: Supplementary Data 3
Description: Figure 3e, f, 4c, d source data.
The absolute abundance of the detected ARGs from the different co-culture systems, including the urban river and Lake West cocultured with *Microcystis aeruginosa* (+Ma) and *Planktothrix agardhii* (+Pa).

File Name: Supplementary Data 4
Description: Total primers of ARG.
